# Effect of Varying Concentrations of Docosahexaenoic Acid on Amyloid Beta (1–42) Aggregation: An Atomic Force Microscopy Study

**DOI:** 10.3390/molecules23123089

**Published:** 2018-11-27

**Authors:** Brenda Yasie Lee, Simon James Attwood, Stephen Turnbull, Zoya Leonenko

**Affiliations:** 1 Department of Biology, University of Waterloo, Waterloo, ON N2L 3G1, Canada; brenda.lee@uwaterloo.ca; 2 Department of Physics & Astronomy, University of Waterloo, Waterloo, ON N2L 3G1, Canada; simonjamesattwood@gmail.com (S.J.A.); s4turnbull@uwaterloo.ca (S.T.)

**Keywords:** atomic force microscopy, docosahexaenoic acid, amyloid, amyloid-beta Aβ, amyloid fibrils, Alzheimer’s disease, omega-3, polyunsaturated fatty acid

## Abstract

Healthcare has advanced significantly, bringing with it longer life expectancies and a growing population of elders who suffer from dementia, specifically Alzheimer’s disease (AD). The amyloid beta (Aβ) peptide has been implicated in the cause of AD, where the peptides undergo a conformational change and form neurotoxic amyloid oligomers which cause neuronal cell death. While AD has no cure, preventative measures are being designed to either slow down or stop the progression of this neurodegenerative disease. One of these measures involves dietary supplements with polyunsaturated fatty acids such as docosahexaenoic acid (DHA). This omega-3 fatty acid is a key component of brain development and has been suggested to reduce the progression of cognitive decline. However, different studies have yielded different results as to whether DHA has positive, negative, or no effects on Aβ fibril formation. We believe that these discrepancies can be explained with varying concentrations of DHA. Here, we test the inhibitory effect of different concentrations of DHA on amyloid fibril formation using atomic force microscopy. Our results show that DHA has a strong inhibitory effect on Aβ_1–42_ fibril formation at lower concentrations (50% reduction in fibril length) than higher concentrations above its critical micelle concentration (70% increase in fibril length and three times the length of those at lower concentrations). We provide evidence that various concentrations of DHA can play a role in the inhibitory effects of amyloid fibril formation in vitro and help explain the discrepancies observed in previous studies.

## 1. Introduction

Alzheimer’s disease (AD) is a common form of dementia primarily observed in the geriatric population with a survival period of approximately eight years after diagnosis [1,2,3]. While physiologically characterized by a huge loss of neurons and disruption of synaptic function in the hippocampus, some physical symptoms include confusion, irritability, memory loss, and eventual loss of bodily functions [3]. With advances in healthcare and longer life expectancies, there are a growing number of individuals who suffer from dementia, making AD a growing concern that is becoming more prevalent in our societies [2]. In fact, it has been projected that hundreds of millions of individuals will be inflicted with this disease by 2050 [4]. Currently, AD is incurable and its cause is still unknown [3,5,6]. However, it is widely accepted that AD is associated with the deposition of amyloid beta (Aβ) in neuronal cells [3,6,7,8,9].

Aβ is a 38–43 residue peptide that is naturally cleaved from the C-terminal region of the amyloid precursor protein (APP), an integral membrane protein that is found within neuronal tissues [8,10,11,12,13]. If proteolytic enzymes cleave the APP abnormally, two neurotoxic fragments known as Aβ_1–40_ and Aβ_1–42_ are produced, with the latter being more neurotoxic than the other as well as being highly implicated in Alzheimer’s disease [8,10,13,14,15]. In AD, the Aβ_1–42_ peptides undergo amyloid fibril formation, a conformational change from alpha-helix to beta-sheet configurations that render the Aβ_1–42_ peptides oligomeric and inclined to form insoluble fibrils and plaques that are deposited in cerebral areas involving cognition and memory [8,16,17,18,19]. However, it is the small soluble Aβ oligomers that are now widely regarded as the most toxic instigators of the neurological damage behind Alzheimer’s disease and should be targeted for therapeutic purposes [20,21,22,23].

Recent studies have focused on dietary components such as polyunsaturated fatty acids (PUFAs) that may help to prevent neurodegenerative diseases such as AD [4,5,24,25,26]. Of these PUFAs, there is an increasing focus on the effects of docosahexaenoic acid (DHA), an omega-3 fatty acid commonly found in fish oils and the central nervous system [25,26,27]. DHA is considered a crucial component of brain development and also a key component of lipids in neuronal membranes where it acts to control neuronal information transfer and modulate the hydrophobicity of the membrane bilayer’s core [24,28,29,30]. Epidemiological studies have also shown that DHA plays a protective role against AD neuropathology, such as the prevention of Aβ aggregation and subsequent inflammatory response [4,28,30,31,32]. Of groups that have observed decreased amyloid fibril formation, one proposed that increased hippocampal neuronal DHA levels would play a more critical role in preventing AD by maintaining neuron survival through the mitigation of Aβ neurotoxicity [33]. Another group suggested that DHA stimulates non-amyloidogenic APP cleavage by proteolytic enzymes, thus reducing the amount of neurotoxic Aβ_1–40_ and Aβ_1–42_ [34]. However, not all studies of DHA and AD have shown that regular or high PUFA supplementation will significantly alter cognitive decline [35]. A possible explanation has been suggested in which the delivery and availability of DHA molecules are believed to be hindered by the permeability of the blood-brain barrier and fatty acid metabolism in the aging brain [36]. A more practical explanation can be attributed to different amounts of DHA included together with nutritional diets, as hypothesized by a group who discovered that different combinations of DHA and other nutrients led to inconsistent results with some diets showing increased amyloid plaques, while others had decreased numbers [37]. Another study indicated that DHA reduced the level of oligomeric amyloid species in a concentration-dependent manner from 0 μM to 20 μM [32]. The beneficial effects of DHA in Alzheimer’s disease still have not been demonstrated through clinical trials, and the level of DHA in AD brains still needs to be clarified [38].

Due to these conflicting results, the question still remains as to whether DHA hinders, promotes, or has no effect on amyloid fibril formation and neural toxicity. In fact, these results lead us to think that varying concentrations of DHA might play different roles in amyloid fibril formation. A study on the molecular level is required to complement the other studies that have been performed thus far to help explain this phenomenon. Atomic force microscopy is widely used to study Aβ aggregation [6,39,40,41,42] and can help shed light onto the mechanism of DHA action on amyloid aggregation.

There have been several atomic force microscopy studies that involve the investigation of individual drugs, molecules, and metals and their effect on Aβ_1–42_ aggregation [12,40,41,43,44]. In fact, recent research by Sublimi Saponetti et al. used atomic force microscopy to show that DHA can act as a membrane-fluidizing agent to help protect the membrane from damage from Aβ_25–35_ peptide aggregates and reduce bilayer defects [45]. Their data indicated that a suitable amount of DHA could act to prevent the toxic action of neurodegenerative proteins such as Aβ in Alzheimer’s disease. However, there has been no study performed on varying concentrations of DHA and its inhibitory effect on amyloid fibril formation using atomic force microscopy.

In this work, we use atomic force microscopy to study the effect of different concentrations of DHA on Aβ_1–42_ fibril formation through a period of 1, 6, and 24 h. The results demonstrated that higher 100 μM concentrations of DHA promoted Aβ_1–42_ fibrillar aggregation, while lower 30 μM concentrations of DHA reduced Aβ_1–42_ aggregation and surface coverage compared to the 0 μM DHA control. Our study complements the previous studies performed on this subject as it studies the phenomenon on a molecular level and helps to explain the differing effect of DHA on amyloid beta formation at different concentrations. This also suggests that DHA could be an important component of dietary and therapeutic measures to prevent the onset or progression of Alzheimer’s disease with the addition of further studies in toxicity.

## 2. Results

**Figure 1:**
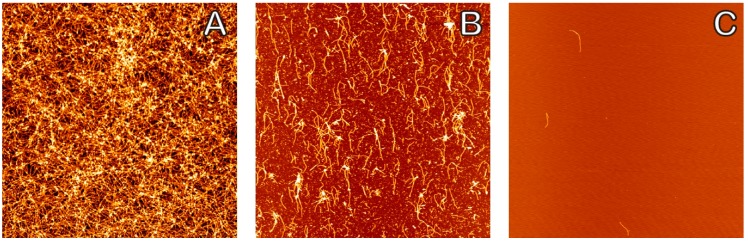
Effect of dilution on amyloid crowding. A comparison of AFM images of amyloid fibrils in the presence of a 100 μM DHA sample at different dilutions, each incubated for 6 h. (**A**) 1:1 dilution showing an extreme number of surface structures and amyloid fibrils crowded together; (**B**) 1:10 dilution with a moderately uniform surface coverage of fibrils that are suitable for image analysis; and (**C**) 1:100 dilution with very few surface features and fibrils. The 1:10 dilution was chosen for further experimentation. The dimensions of each image are 10 × 10 μm.

Atomic force microscopy was used to capture the quantity and development of Aβ_1–42_ oligomers, fibrils, and larger aggregates in the presence of DHA at varying concentrations. It was previously determined that the critical micelle concentration (CMC) of DHA is approximately 60 μM [46]. We used three different concentrations: A 0 μM DHA sample as the control for typical amyloid fibril formation, a 30 μM submicellar DHA concentration, and a 100 μM supramicellar DHA concentration to understand the effect that different amount of DHA would have on amyloid aggregation. Each sample was diluted by a factor of 10 to optimize the distribution and amount of aggregates for imaging and analysis. Figure 1 shows AFM topography images of these fibrils at dilutions 1:1 (1A), 1:10 (1B), and 1:100 (1C). The 1:10 dilution resulted in less crowding of the fibrils (see Figure 1B), as compared to overcrowding with no dilution and few visible fibrils in the 1:100 dilution samples.

**Figure 2:**
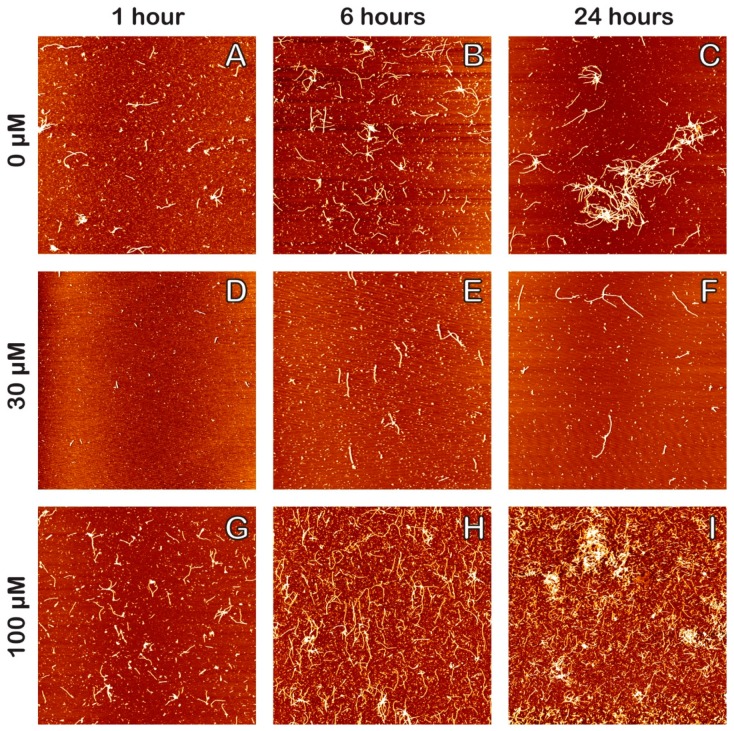
Atomic force microscopy images of Aβ_1–42_ fibril formation with varying concentrations of DHA at different incubation times. Aβ_1–42_ with 0 μM DHA (**A**–**C**), 30 μM DHA (**D**–**F**), and 100 μM DHA (**G**–**I**) were incubated at 1, 6, and 24 h, respectively (images from left to right). To prepare the samples, 50 μL aliquots of each solution were deposited onto freshly cleaved mica for 5 min, washed with Milli-Q nanopure water, and dried with nitrogen gas after their respective incubation times. The concentration of Aβ_1–42_ in each of the samples is 110 μM and the dimensions of each image are 10 × 10 μm.

Figure 2 shows representative AFM images of amyloid fibrils formed in solution with 0, 30, and 100 μM DHA, each incubated at 1, 6, and 24 h to give a total of nine different samples. Qualitatively, we can see that each set of images for different DHA concentrations possesses a general trend where longer incubation times lead to an increase in the number and length of Aβ_1–42_ oligomers and fibrils. Compared to the control samples for 0 μM DHA (Figure 2A–C), we qualitatively observe that there are fewer oligomers and fibrils in each of the 30 μM DHA samples (Figure 2D–F). Meanwhile, the 100 μM DHA samples (Figure 2G–I) were revealed to have a large, growing network of long fibrils and aggregates with increased surface coverage, as compared to the control.

**Figure 3:**
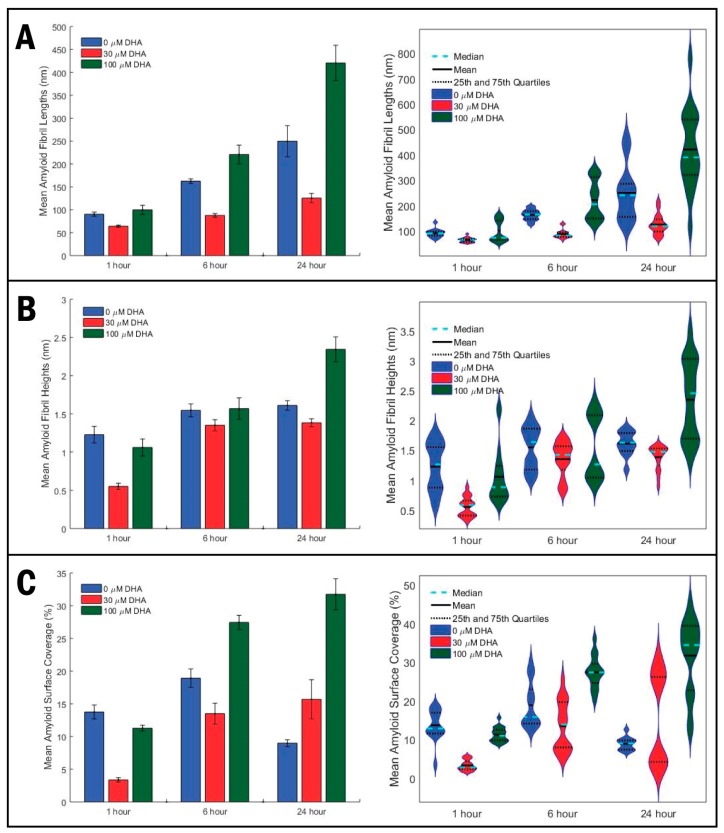
Statistical analysis of amyloid fibril length, height and surface coverage. For comparison, we presented statistical analysis of AFM topography images in two ways: as bar graphs (left column) and violin plots (right column). The mean values of Aβ_1–42_ fibrils were compared between three concentrations of DHA (0, 30, 100 μM) at incubation times of 1, 6, and 24 h. Mean values of fibril lengths (**A**), fibril heights (**B**), and surface coverage (**C**) were obtained through image processing using MATLAB code, where values were measured for at least eight images for each repeat performed. The variance of our data is represented with violin plots in the right column, where the mean, median, and interquartile range are presented (refer to legends), as well as the data distribution and density (refer to widths for frequency of data).

Figure 3 shows the results of statistical analysis of amyloid fibril length (3A), height (3B), and surface coverage (3C) obtained from AFM topography images, presented as bar graphs in the left column, and violin plots in the right column. Figure 3A shows the averaged mean fibril lengths for the AFM topography images obtained for each of the nine samples. As observed in AFM images in Figure 2, each set of samples for different DHA concentrations have increasing amyloid fibril lengths as incubation time progresses from 1 to 6 to 24 h. From Figure 3A–C, we can see that with the 30 μM DHA data set, fibril length gradually increases after 6 h from 64 ± 2 nm to 88 ± 4 nm, and again after 24 h to 126 ± 10 nm. However, this final value is significantly less than the control (no DHA) whose mean fibril length is 250 ± 34 nm after 24 h, almost double that of the 30 μM sample. The 100 μM DHA data set yields considerably longer fibril lengths and greater variance than the other DHA concentrations at every incubation time, which coincide with the tangled network of long fibrils observed in Figure 2G–I. Its final fibril length at 24 h was 421 ± 38 nm, well over triple the length of the 30 μM DHA sample. A similar trend can be seen with the averaged mean fibril heights for each sample (Figure 3B), where fibril heights gradually increase as time progresses and the 100 μM DHA samples having the highest values at 24 h once again. Figure 3C represents the statistical analysis of the surface coverage of Aβ_1–42_ for the sample image. It can be reasoned that there is significantly more surface coverage with 100 μM DHA versus both the control and 30 μM DHA, while the 30 μM DHA has lesser surface coverage for both the 1 and 6 h incubation times.

These results (Figure 2A–C) show that, compared to the control (0 μM DHA samples), a low concentration (30 μM) of DHA seems to moderately inhibit this process, as seen by shorter fibril lengths and heights as well as a reduced amount of surface coverage (Figure 2D–F). Meanwhile, a much higher concentration of DHA (100 μM DHA) shows, without a doubt, that amyloid fibril formation is promoted due to much longer fibril lengths, heights, and extensive surface coverage, Figure 2G–I.

## 3. Discussion

The goal of this study was to use atomic force microscopy to elucidate the inhibitory effect of DHA on amyloid aggregation on a molecular level. This has not been shown before in the context of studying the sole effect of DHA on amyloid beta aggregation.

Though no cure to Alzheimer’s disease has been discovered, there is an unending effort to identify and slow down the progression of the disease, such as the use of inhibitors and dietary supplements. Current medications are mainly acetylcholinesterase inhibitors that target the treatment of AD-associated cognitive problems and symptoms, but they have been found to be of little use or benefit [47,48,49]. As a result, the focus has now been shifted towards preventing AD at the source: Amyloid fibril formation. All aggregation processes must first start with the dimerization of two molecules that spark the pathway to further conformational developments that produce additional and larger aggregated structures. In the case of Aβ and Alzheimer’s disease, it would be ideal if we could stop the initial formation of the dimer completely. Numerous studies have been performed with the use of atomic force microscopy to elucidate the interactions between different biomolecules and their inhibitory effects on amyloid fibril formation [40,41,50,51,52,53,54,55,56]. In experiments from Kawashima and colleagues, Aβ_1–42_ was modified at Gly(25)-Ser(26) and its inhibition of amyloid fibril formation was verified using AFM images [51]. Previously, we studied the effect of biometals and synthetic inhibitors, where AFM images were also taken of amyloid fibrils at time intervals of 1, 6, and 24 h [40,41]. With these papers, their results coincided with ours for the Aβ control (or 0 μM DHA samples) in terms of increasing fibril length and height as time progressed.

Recent studies and reviews have provided conflicting results as to whether DHA alone could be useful in preventing and treating cognitive decline for patients with Alzheimer’s disease [31,38]. A topic of interest involves the role that DHA plays in mediating the processing of the amyloid precursor protein and amyloid fibrillation. Fairly consistent results have been shown with DHA reported to have ameliorated amyloid levels by restraining toxic Aβ fragmentation and fibrillation [32,34,57,58], but other reports that involve human studies have shown that DHA has no effect on such processes and may instead aggravate them [31,37,59]. A recent study examined such mixed results in patients at risk of developing AD (with the apolipoprotein E ε4 (*APOE4*) allele), suggesting that the interactions between DHA, the *APOE4* genotype, and the stage of AD pathology could account for the different results observed from different studies [60]. Their hypothesis is in line with ours, where the incorporation of DHA supplementation can result in beneficial outcomes if the timing and dosage are correct. However, this study did not examine the immediate effect of DHA on amyloid fibril formation at different concentrations.

When DHA was shown to have an inhibitory effect on amyloid aggregation, it was hypothesized that it promotes the non-amyloidogenic processing of the amyloid precursor protein and reduces the levels of Aβ in the brain [14,34,61]. A study by Hashimoto et al. observed that Aβ_1–40_ oligomers decreased in a concentration-dependent manner from 0 to 20 µM DHA. Their study focused solely on DHA, and their results suggested that DHA alone could both inhibit the deposition and formation of Aβ fibrils [57]. A different study was performed on lipid bilayer models with and without 20% DHA composition [45]. Using atomic force microscopy and both dry air and liquid imaging techniques, the group deduced that DHA can both protect the membrane from peptide aggregates and reduce defects in the bilayer during the process of delipidation. Clearly, past research has shown that there are beneficial and inhibitory effects of DHA on amyloid fibril formation. Our results for the submicellar 30 μM DHA samples correlate with such hypotheses, showing decreased fibril length, height, and surface coverage as time progressed. This led us to the conclusion that submicellar DHA concentrations could inhibit amyloid fibrillation and reduce amyloid levels.

On the other hand, supramicellar DHA concentrations were observed to produce the opposite effect upon analysis of our AFM images, resulting in much greater fibril lengths, heights, and surface coverage than both the control and 30 μM DHA samples. The study from Hashimoto et al. studied varying DHA concentrations up to and including 20 µM of DHA and showed decreasing amyloid content [57]. Our results correlate with this trend up until the critical micelle concentration of DHA is surpassed at 100 µM, where the opposite trend was observed. This suggests that at some threshold, DHA stops conferring inhibitory effects. A clinical trial by Freund-Levi et al. aimed to study the effect of DHA on mild to moderate AD subjects using DHA, EPA (eicosapentaenoic acid), or a placebo [62]. The mini mental state examination and AD assessment scale scores were indifferent between each of these groups despite daily doses of DHA (1.7 g) that were many times higher than typical DHA content in fish supplement products [62]. Another trial by Quinn et al. used a dosage of 2 g of algal DHA per day for 18 months and found no effect on the rate of cognitive decline in AD patients [35]. Increased levels of neuronal loss and prion formation have also been reported in cell-culture AD models following DHA supplementation [63]. A study by Amtul et al. has also been performed on DHA supplementation combined with other lipids or nutrients. When taken with peptamen, DHA not only has no effect on amyloid plaque build-up, but may even spur the production of Aβ peptides and plaques in transgenic mouse models [37]. These results correlate to our results for higher DHA concentrations.

Our data cover both lower and higher concentrations of DHA, and our results correlate well with previously reported data for each submicellar and supramicellar concentration range. In both concentration ranges, AFM topographical imaging was used to test their effect on amyloid fibril formation, where the addition of DHA triggered a structural change in the fibrils observed, both qualitatively and quantitatively. Typical Aβ oligomers will form fibrils by growing in length and height, subsequently leading to the formation of fibrillar aggregates and plaques through AD pathogenesis. In comparing the 30 μM DHA AFM images to the control (see Figure 2), we observe shorter fibrils for each incubation time (see Figure 3A–B), indicating that DHA inhibits the polymerization of oligomers to fibrils. The small error associated with both the mean fibril length and height emphasize the consistency of our results. The surface coverage was also analyzed for each AFM image (Figure 3C), and with the 30 μM DHA samples, this parameter increased at a slower rate than the control. Our analysis shows that submicellar concentrations of DHA play a role in inhibiting the progression of amyloid fibrillations.

Our data are different between the supramicellular 100 μM DHA and submicellar 30 μM DHA concentrations. In comparison to the control, the 100 μM DHA samples had consistently greater mean fibril lengths, heights, and surface coverage, with values doubling those of the control in some cases (Figure 3). In the AFM images shown in Figure 3G–I, it can be seen that with 100 μM DHA, the oligomers grow into fibrils fairly quickly, with many of them showing up at 6 h at extended lengths. At 24 h, we can see a tangled network of fibrils, and among them, some denser areas that we believe to be the development site of amyloid plaques.

Based on our data and previously published studies, we hypothesize that DHA can be beneficial and inhibit the progression of amyloid fibrillation at submicellar concentrations, but can induce fibril formation and elongation at supramicellar concentrations. We believe that this hypothesis can explain the discrepancies observed in previous studies with conflicting results, where DHA either inhibited, promoted, or had no effect on amyloid fibrillation. In several studies, it was reported that DHA reduced Aβ levels in either cell cultures or AD animal models [32,61,64,65,66]. Sahlin et al. proposed that this phenomenon could be explained by DHA’s effect on altering APP processing so that reduced Aβ levels would be observed [34]. Other studies show that DHA either has no effect or a detrimental effect in amyloid beta fibril formation [35,37]. In Reference [37], DHA was investigated with two dietary regimes, one of which was a peptamen and DHA diet. The mice on this diet showed a strong preference in producing Aβ oligomers and plaques [37]. In Reference [35], the supplementation of DHA compared with a placebo resulted in no difference in the rate of cognitive or functional decline in AD patients, which did not support the hypothesis that solely DHA supplementation would slow down or prevent the progression of AD [35]. Since both of these studies focused on the effect of DHA alone on amyloid beta, it further strengthens our case that DHA can have both a positive or negative effect on amyloid fibril formation depending on its concentration. Our work covers a wide range of concentrations of DHA used, and provides a plausible link between previously contradictory studies.

In conclusion, we demonstrate that DHA alone can in fact inhibit the progression of amyloid fibril formation, but only in small regulated amounts. Should an excess of DHA be present, the opposite effect takes place in which amyloid fibril lengths, heights, and surface coverage increase drastically compared to the control and 30 μM DHA samples. However, even though we have shown these trends, several questions remain unanswered, primarily involving the exact quantity of DHA that is the tipping point between inhibition and promotion of amyloid fibril formation as well as how that correlates to suggested amounts of dietary supplements of DHA, either as a preventative measure for Alzheimer’s disease (or other neurodegenerative diseases involve Aβ_1–42_) or as part of a treatment therapy or mixture of dietary supplements for those already diagnosed with this disease. This interesting behavior of DHA at different concentrations should be taken into account when considering the relation between dietary concentrations compared to local concentrations in the brain, in order to find an optimum concentration of DHA for the inhibition of amyloid fibril formation. Additional studies should also be performed to elucidate the effect that DHA, and possibly other fatty acids, have on amyloid fibril formation in different physiological conditions pertaining to each stage of Alzheimer’s disease, from health neuronal cells to damaged neuronal cells.

## 4. Materials and Methods

To elucidate the effect of docosahexaenoic acid (DHA) on amyloid beta fibril formation, three concentrations of DHA (0, 30, 100 μM) were incubated with Aβ_1–42_ for three different time intervals (1, 6, 24 h). A JPK NanoWizard II atomic force microscope (AFM) (JPK Instruments AG, Berlin, Germany) was used to obtain images of Aβ_1–42_ oligomers and fibrils to be analyzed. In an AFM’s imaging mode, a sharp probe or tip on a cantilever is rastered across the surface of an atomically flat substrate such as mica. As the surface is scanned, forces between the tip and sample cause the cantilever to deflect. This deflection is measured by a laser reflecting off the back of the cantilever and onto a photodiode, resulting in a high-resolution image of the sample’s surface topography [67].

### 4.1. DHA and Aβ_1–42_ Incubation & Sample Preparation

DHA was stored at −20 °C after being purchased from Cayman Chemical Company (Ann Arbor, MI, USA) as a solution immersed in ethanol with ≥98% purity. A 20 mM HEPES and 100 mM NaCl buffer solution was adjusted to a pH of 7.4 and filtered through a 0.2 μm membrane. This buffer was then used to prepare a 0.75 mM DHA stock solution.

Aβ_1–42_ was purchased from rPeptide in lyophilized powder form, in monomeric form, and pretreated according to Fezoui protocols [68]. The HEPES buffer was used to create an Aβ_1–42_ solution with an initial concentration of 625 μg/mL. This solution was immediately divided into three equal aliquots so that different ratios of DHA stock solution and HEPES buffer could be added to each sample (with final Aβ_1–42_ concentration of 500 μg/mL or 110 μM) to result in three classes of DHA concentrations: 0 μM (control), 30 μM (submicellar or below CMC), and 100 μM (supramicellar or above CMC). Each of the DHA/Aβ_1–42_ samples was incubated for 1 h, 6 h, and 24 h at 37 °C for a total of nine samples.

After each incubation time, calculated proportions of each DHA/Aβ_1–42_ solution and distilled H_2_O were homogenized in a plastic centrifuge tube so as to dilute the sample by a factor of ten. It is important to note that from past experiments, a clear trend was noticed within each of the various concentrations of DHA. However, there were difficulties in obtaining images for analysis because the 1:1 and 1:2 dilutions resulted in too many fibrils and aggregates on the surface of the mica once imaged, and the 1:100 dilutions resulted in too few fibrils and aggregates. Only the 1:10 dilution images had a moderate amount of fibrils that rendered it more suitable for image analysis. As a result, the 1:10 diluted DHA/Aβ_1–42_ solution was deposited onto a freshly cleaved mica slide, with each sample having a total deposition of 50 μL, incubated on mica for 5 min, and then rinsed six times with 50 μL of distilled H_2_O at an approximate 45° tilt to remove any unbound structures or salt crystals on the surface of the mica that may form from the buffer solution. A gentle stream of nitrogen gas was applied to each mica sample for two minutes until fully dried. At least three samples were made for each concentration in every trial.

### 4.2. Atomic Force Microscopy Imaging

The JPK Nanowizard II atomic force microscope was used to image each mica slide with Aβ_1–42_. Aggregates formed in the presence of 0, 30, or 100 μM DHA and adsorbed onto the surface of the mica slide. The NCH AFM cantilevers were purchased from NanoWorld (Neuchâtel, Switzerland), designed for non-contact and tapping mode imaging to offer high sensitivity and speed while scanning (320 kHz resonance frequency, 42 N/m force constant, thickness 4 μm, no coating). 10 × 10 μm and 5 × 5 μm images were taken in air in Intermittent Contact mode. The experiments were repeated at least six times. The trials at 1:10 dilutions were used for statistical analysis, with at least eight 10 × 10 μm images obtained for each sample at a resolution of 1024 pixels; approximately 5000 data points were taken from each sample.

### 4.3. Image Analysis

**Figure 4:**
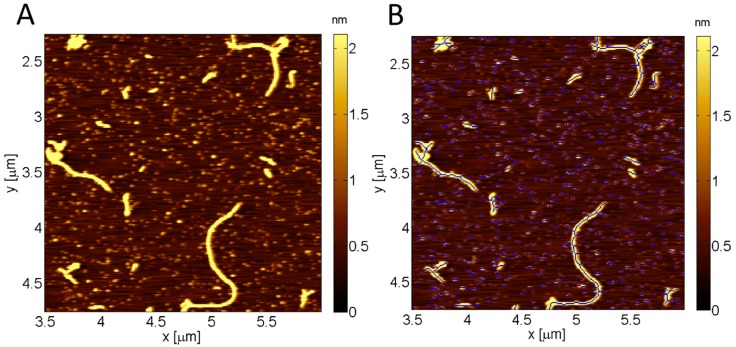
Image analysis using custom MATLAB code to measure the length, height and surface coverage of fibrils. (**A**) AFM topography image of amyloid fibrils and oligomers on a mica surface; and (**B**) custom-developed routine in MATLAB successfully detects and traces the long axis of all fibrils and oligomers, as shown in blue.

All image analysis was performed using custom developed routines written in MATLAB (R2016a, MathWorks, Natick, MA, USA). Figure 4 illustrates the application of this MATLAB routine to a sample AFM topography image, and represents the image before (4A) and after (4B) this routine is applied. Each image was initially plane flattened, line-by-line flattened, and color scales adjusted as necessary. Based on user input, an arbitrary threshold was conducted on the entire image with the aim of excluding all topographical features above the background substrate. Using the mask created beforehand, the image was flattened using a row-wise second order polynomial subtraction. By excluding the highest topographical features first before conducting the polynomial flatten, artefacts are minimized in the background subtraction. A Gaussian distribution was then fitted to the histogram of pixel heights using the Levenberg-Marquardt non-linear least squares routine for all pixels associated with the background (i.e., the masked image). The peak of the distribution represents the average of the background and thus all other height measurements are made relative to this value. This is much more accurate than measuring local height changes that depend strongly on the background noise. A user-defined threshold was then conducted on the entire flattened image to exclude the background and highlight only the pixels associated with the features of interest. A search algorithm was employed to find each separate feature (fibril or oligomer) by considering pixel clusters that were separated from other clusters by pixels representing the background. In this simple approach, some fibrils or oligomers that were close to each other or overlaid in the image would be considered as a single object, so the length parameter may, in some cases, have been overestimated. This problem cannot easily be resolved, however the same approach is applied to all samples so, although absolute values may vary from reality, the trends in differences between samples are reliable. If a fibril were observed to have branching (either due to outgrowths from the main fibril or due to overlaid fibrils) then it would still be counted only as one fibril and the length of that fibril would be equal to the combined length of all the component branches (Figure 4B).

Another concern was that since the histogram of pixel heights associated with the fibrils often overlapped the histogram of pixels heights for the background (i.e., both the background level and the fibril level have considerable height fluctuations that overlap), there was a necessary judgement by eye that must be made and cannot easily be automated or defined completely objectively. However, we believe that by averaging over many images and different samples, this effect would simply be represented as a contribution to the final uncertainty in our measurements.

In turn, each identified feature or cluster of pixels was then analyzed to find the length of its long axis. We arbitrarily defined a fibril to be a feature that has a length at least twice that of its width. The surface area was calculated as the percentage of pixels identified in the thresholding relative to the total number of pixels. Thus, percent coverage included contributions from both fibrils and oligomers. For the fibril height calculation, the maximum pixel height within the cluster of pixels corresponding to each fibril was taken and the background height subtracted. We could have chosen to average the height of all pixels for a given fibril rather than taking the maximum, but this would have underestimated the maximum height of the fibril. The method we have used could be prone to local pixel noise, however very little noise of this kind was observed on the fibrils for all images, so we believe it is an appropriate approach to take.

In summary, for image analysis, the preferred experimental conditions were as follows: A concentration of 500 μg/mL or 110 µM Aβ_1–42_ diluted to 1:10 in water, and aliquots of 50 µL placed on mica and incubated for 5 min before rinsing and drying. This preparation allows for an optimal density of fibrils and aggregates so that they can be individually resolved and analyzed.

### 4.4. Statistical Analysis

Statistical analysis was performed to determine the mean amyloid fibril lengths, heights and surface coverage across different samples and time incubations. At least eight images for each trial were analyzed, with about 5000 measurements per image. The results of this statistical analysis were presented as both bar graphs and violin plots to show variance. Bar graphs and violin plots were created using MATLAB. For violin plots, modified MATLAB code was created using the function written by Hoffmann H, 2015: *violin.m—*simple violin plot using MATLAB default kernel density estimation (INRES, University of Bonn, Katzenburgweg, Germany).
